# Percutaneous aspiration mechanical thrombectomy in a 14-year-old with acute limb ischaemia post coarctation of aorta stenting: a case report

**DOI:** 10.1093/ehjcr/ytaf321

**Published:** 2025-07-22

**Authors:** Raja Muhammad Burhanudeen Afiq Raja Badrol Hisham, Mathan Mohan J. Munusamy, Ismazizi Zaharudin, Geetha Kandavello

**Affiliations:** Department of Cardiothoracic and Vascular Surgery, Institut Jantung Negara, Malaysia; Paediatric and Congenital Heart Centre, Institut Jantung Negara, Malaysia; Department of Cardiothoracic and Vascular Surgery, Institut Jantung Negara, Malaysia; Paediatric and Congenital Heart Centre, Institut Jantung Negara, Malaysia

**Keywords:** Percutaneous aspiration thrombectomy, Mechanical thrombectomy, Minimally invasive peripheral thrombectomy, Acute limb ischaemia, Computer-assisted vacuum thrombectomy, Case report

## Abstract

**Background:**

The treatment of vascular diseases has evolved significantly over the years, with recent advancements in interventional strategies, particularly in the management of acute limb ischaemia (ALI). Endovascular and catheter-based interventions, including aspiration mechanical thrombectomy, are now commonly employed and well-documented in the adult population. However, their use in paediatric patients remains rare and underreported. This case report presents our experience managing ALI in a paediatric patient using an endovascular approach—a relatively novel technique in this population with limited data currently available.

**Case summary:**

We report our experience managing a case of ALI in a 14-year-old girl who underwent an elective stenting procedure for coarctation of the aorta. Although the primary intervention was completed, the patient subsequently developed thrombosis of the left common femoral artery. She was treated with aspiration mechanical thrombectomy using a computer-assisted vacuum thrombectomy system. The procedure was successful, resulting in prompt re-establishment of lower limb perfusion and a favourable clinical outcome.

**Discussion:**

This case report offers anecdotal evidence that may serve as a catalyst for future studies evaluating the feasibility of an endovascular approach using aspiration mechanical thrombectomy in the paediatric population. It highlights the potential to identify a clearly defined subset of patients who may benefit most from such interventions. Our experience provides valuable clinical insight and adds to the growing armamentarium for managing ALI in young children.

Learning points

- Data on endovascular approach for treatment of acute limb ischaemia in paediatric populations are still lacking.
- Endovascular approach of aspiration mechanical thrombectomy for paediatric population is feasible with suitable anatomy.

## Introduction

The management of acute limb ischaemia (ALI) in children remains a topic of significant contention due to the relative rarity of such events compared to the adult population.^1^ There has been an increasing use of endovascular interventions, including aspiration mechanical thrombectomy. An effective approach to ALI must be comprehensive, considering various factors such as aetiology, patient age, body weight, vessel size, and other relevant considerations. Here, we report our experience managing ALI in a 14-year-old child using aspiration mechanical thrombectomy with a computer-assisted vacuum system.

## Summary figure

**Figure:**
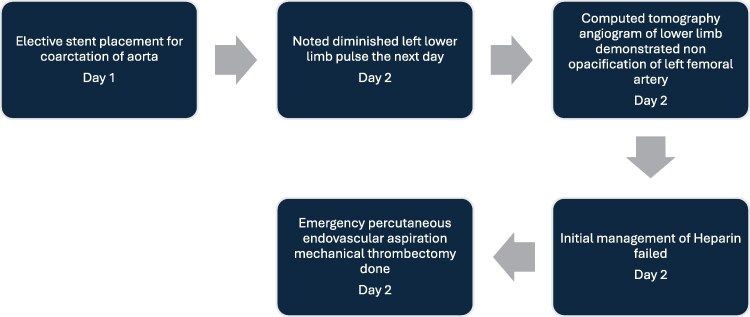


## Case presentation

A 14-year-old girl with underlying Takayasu arteritis, dilated cardiomyopathy, and coarctation of the aorta was electively admitted for stenting of the coarctation. Pre-procedural echocardiography and cardiac magnetic resonance imaging revealed a left ventricular ejection fraction ranging from 16% to 25%, with no evidence of left ventricular thrombus.

**Figure 1:**
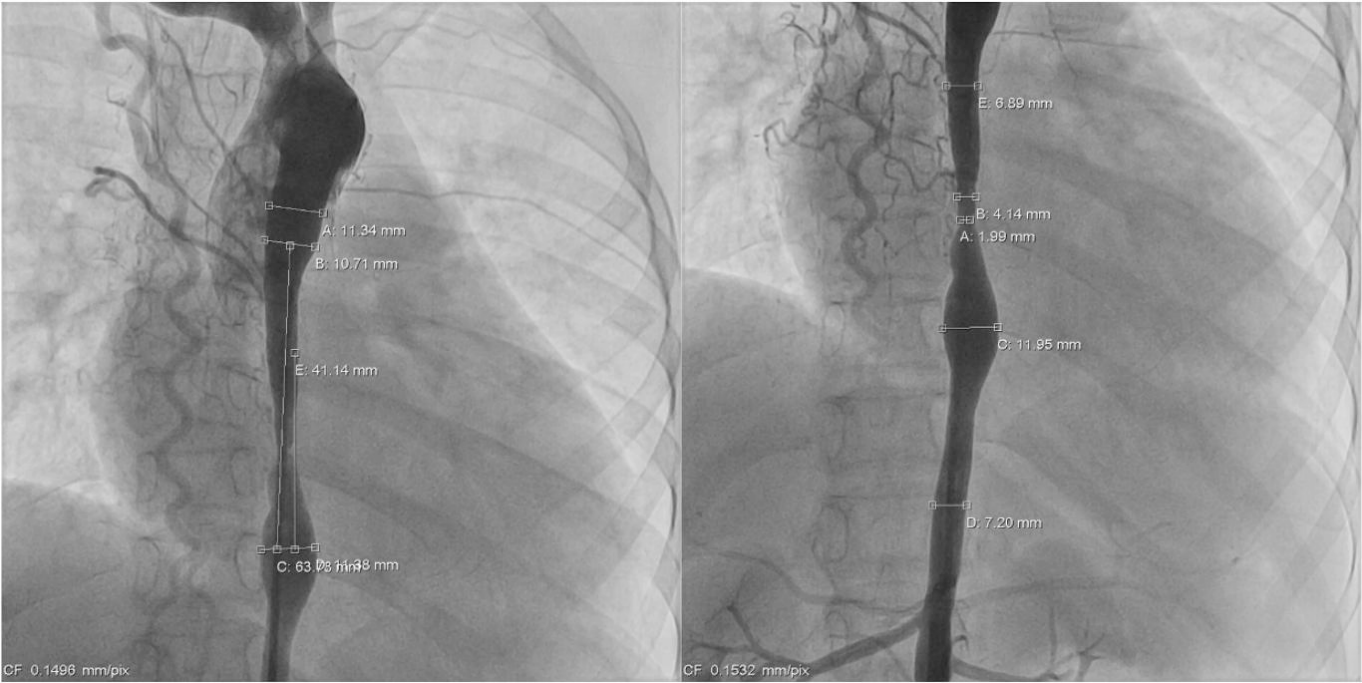
The pre-stenting coarctation of aorta.

**Figure 2:**
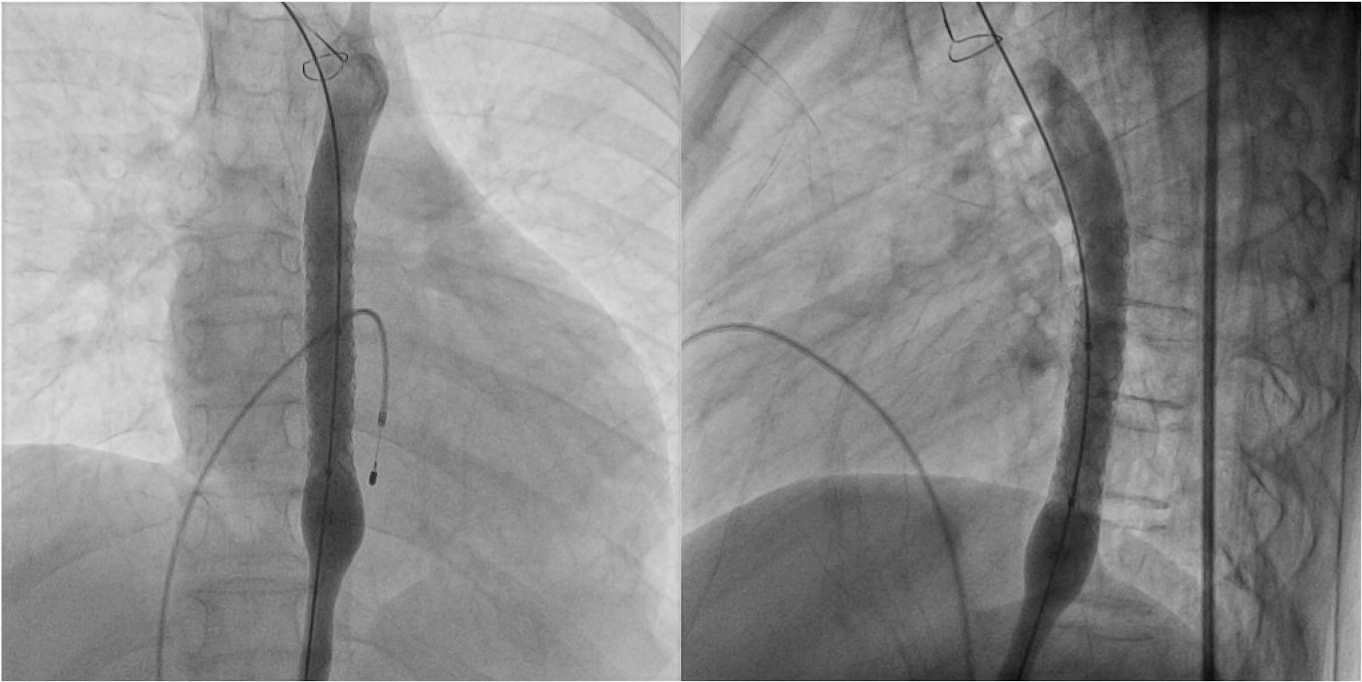
Final result after coarctation stenting showing good placement of stent with no dissection.

The procedure was performed via access through the right femoral artery (RFA), left femoral artery (LFA), and right femoral vein, each using a 6 Fr sheath. Pre-stenting angiographic imaging revealed severe coarctation at the T6–T7 level, with the narrowest diameter measuring 1.99 mm. The lesion exhibited a long, hourglass-shaped narrowing, with the coarcted segment measuring 63 mm in total length (*Figure 1*). Balloon angioplasty of the coarctation was first performed using a 10 × 40 mm Armada balloon catheter. Subsequently, stenting was achieved with overlapping 10 × 39 mm and 10 × 29 mm Palmaz Genesis stents (*Figure 2*). Activated clotting time (ACT) was maintained above 300 s throughout the procedure with intravenous heparin. During deployment, brief embolization of the second stent towards the proximal descending aorta occurred and required retrieval using a snare introduced via the right axillary artery (8 Fr short sheath). Both stents were eventually deployed successfully at the intended site and remained stable, and post-stent angiography demonstrated significant resolution of the coarctation.

The RFA sheath was removed and closed uneventfully using the PerClose ProGlide system. However, haemostasis could not be achieved at the LFA access site with the initial closure attempt, necessitating the deployment of a second percutaneous suture device to control bleeding. Distal lower limb pulses were palpable and unremarkable immediately following the procedure.

**Figure 3:**
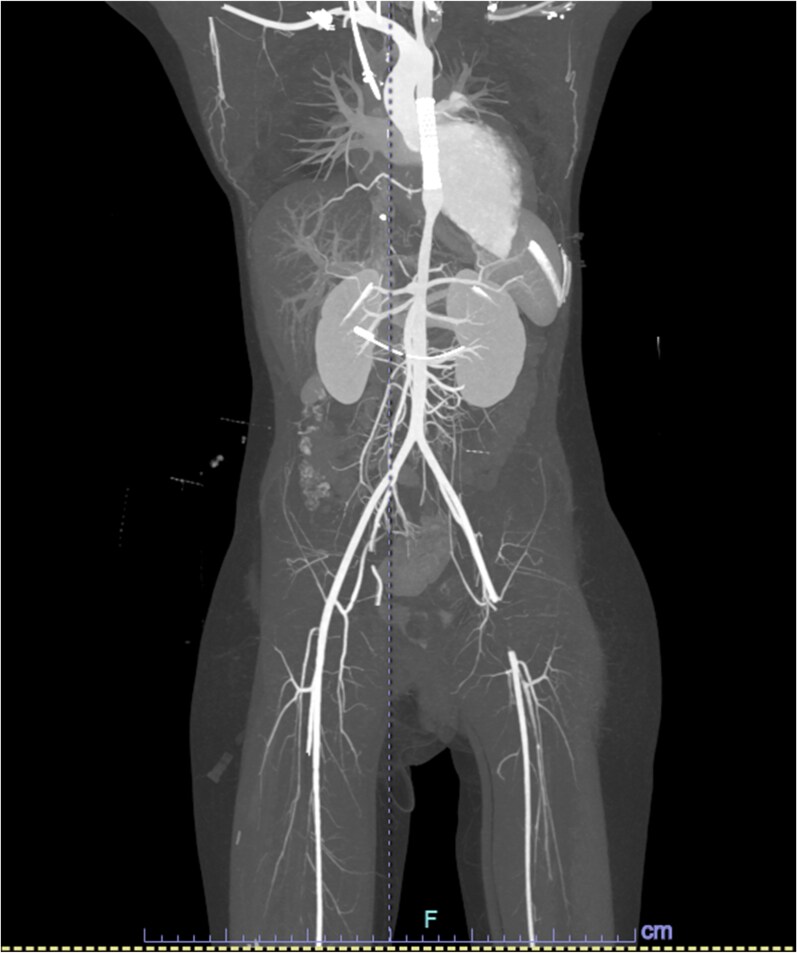
The reconstructed computed tomography angiogram images (pre-aspiration mechanical thrombectomy) showing thrombosis of left common femoral artery, with visible aortic stents after coarctation of aorta stenting.

**Figure 4:**
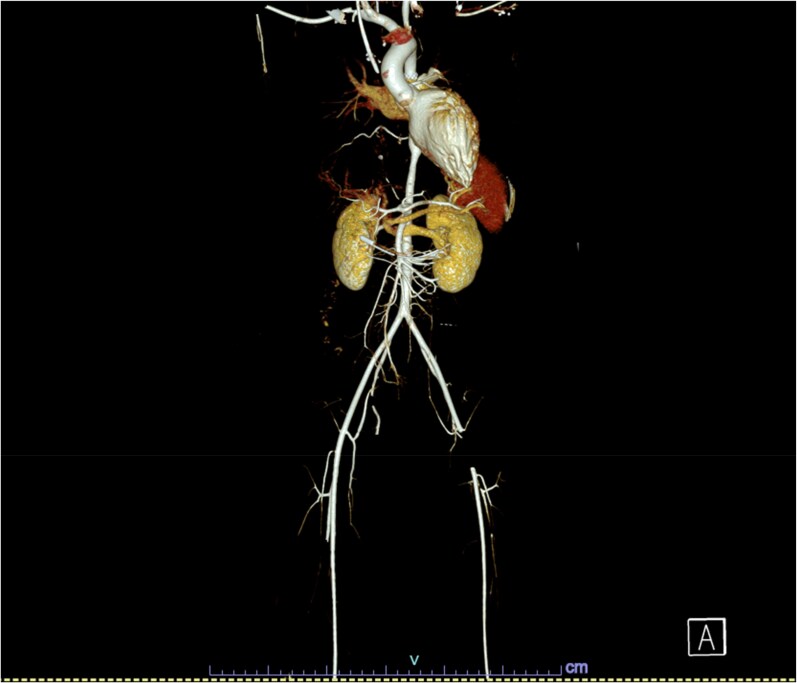
The 3D reconstruction of computed tomography angiogram images (pre-aspiration mechanical thrombectomy) showing thrombosis of left common femoral artery, with visible aortic stents after coarctation of aorta stenting.

On the following day, diminished pulses were noted in the left lower limb, and there were no associated neurological deficits. Computed tomography angiography of the lower limbs demonstrated a long segment of non-opacification of the LFA as shown on the reconstructed computed tomography angiogram (*Figures 3* and *4*). Initial management with unfractionated heparin yielded no clinical improvement. The case was subsequently referred to the vascular surgery team, and the decision was made to proceed with emergency left lower limb angiography and mechanical thrombectomy.

**Figure 5:**
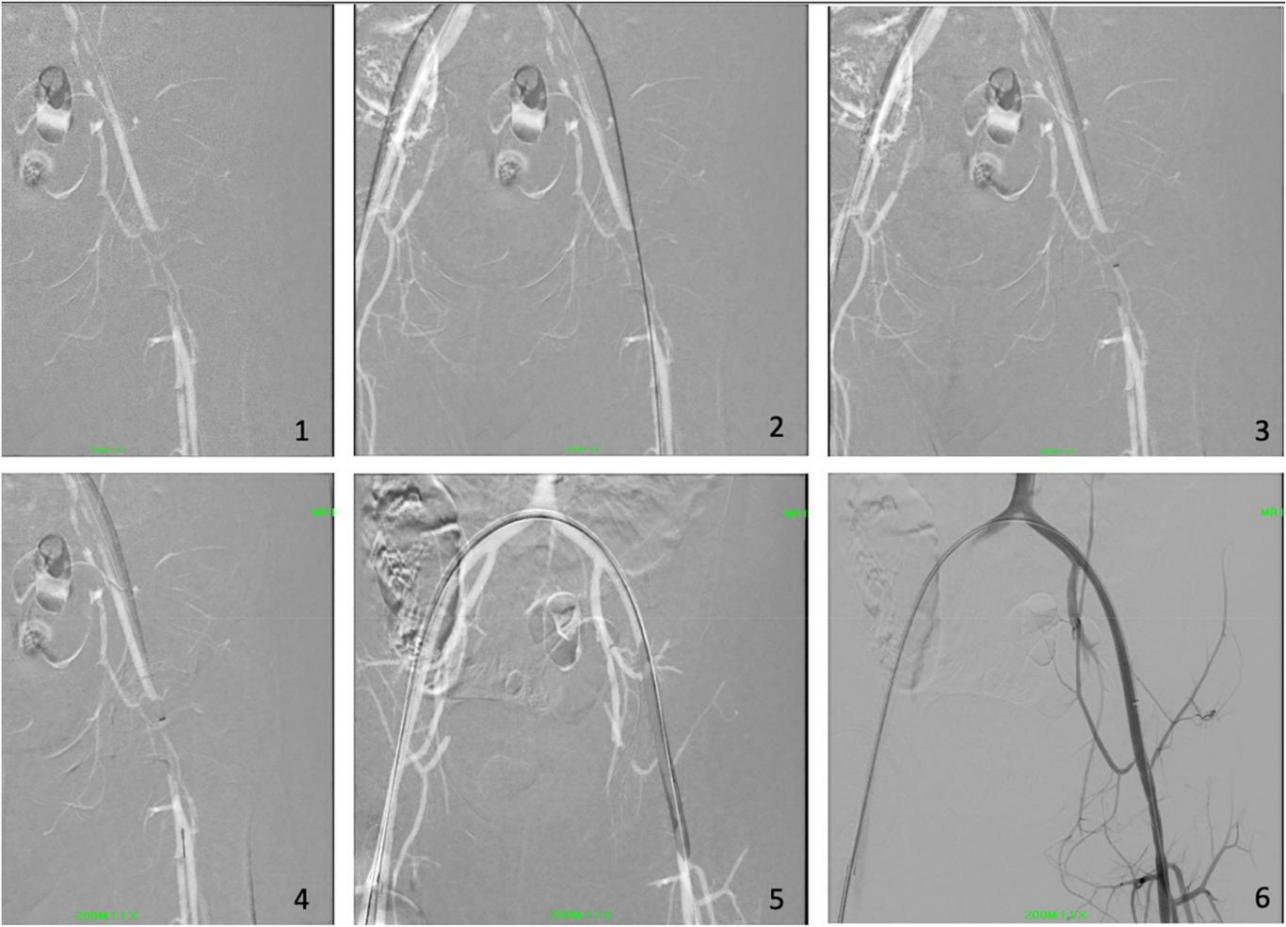
Fluoroscopic images of endovascular aspiration mechanical thrombectomy. (1) Initial lesion on left common femoral artery. (2) Able to cross the lesion using guidewire. (3) Aspiration mechanical thrombectomy device deployed. (4) Embolic protection device placed beyond the thrombosis. (5) Balloon angioplasty done. (6) Completion Angiography was performed after the procedure.

Angiography revealed complete thrombosis extending from the left external iliac artery to the left common femoral artery (CFA). Before thrombectomy, a single dose of low molecular weight heparin at 1 mg/kg was administered. During the procedure, a bolus followed by a continuous infusion of unfractionated heparin was given by institutional protocol, with ACT maintained between 180 and 200 s. Completed procedural angiography is shown in *Figure 5*.

Percutaneous access was obtained via the RFA, and the PerClose ProGlide™ SMC system was applied. A guidewire was successfully advanced across the thrombosed segment, followed by the insertion of a 7 Fr sheath. An aspiration thrombectomy catheter was introduced through the sheath, and an embolic protection device was positioned distal to the thrombus. Mechanical thrombectomy was then performed using the standard protocol with a computer-assisted vacuum thrombectomy system (Penumbra Indigo® Aspiration System with Lightning), followed by balloon angioplasty. Balloon angioplasty was carried out to address minimal residual stenosis, with the aim of improving vessel patency and reducing the risk of re-occlusion. Estimated blood loss was approximately 50 mL. Post-procedural angiography demonstrated good flow, which was confirmed with intravascular ultrasound. A small, non-flow-limiting dissection was noted at the left CFA, likely at the site of the previous puncture. Lower limb perfusion was restored, and distal pulses were well-palpable following the procedure.

## Follow-up

The patient was discharged well on dual antiplatelet therapy and was ambulatory with preserved distal pulses and adequate perfusion of the lower limb. Given that the ALI was attributable to maldeployment of the PerClose ProGlide™ system rather than an intrinsic coagulopathic process, anticoagulation was deemed unnecessary. Although single antiplatelet therapy is the institutional standard of care following stenting procedures, a precautionary 3-month course of dual antiplatelet therapy was initiated to mitigate the risk of recurrent thromboembolic events. At the 3-month follow-up, the patient was transitioned to antiplatelet monotherapy, which is anticipated to be continued lifelong due to the underlying diagnosis of Takayasu arteritis and its associated high propensity for neointimal hyperplasia.

## Discussion

Acute limb ischaemia is uncommon in the paediatric population and exhibits a distinct aetiological profile compared to adults. Single-centre studies have demonstrated that most paediatric ALI cases involve the lower extremities and are predominantly attributable to catheterization-related^2^ vascular injury, as exemplified in this case. The use of the PerClose™ vascular closure device has been associated with vessel occlusion as a recognized complication.^3^ Management of this case was further complicated by underlying comorbidities, including autoimmune pathology and dilated cardiomyopathy with reduced ejection fraction, both of which may predispose to thrombogenesis. Therefore, deployment of the PerClose™ device warrants cautious consideration in patients presenting with such risk factors, given the potential for these conditions to exacerbate thrombotic events.

Previous consensus statements and clinical studies indicate that anticoagulation and thrombolysis are safe and effective modalities for the management of ALI in the paediatric population,^4^ with invasive interventions typically reserved for cases refractory to initial therapy.

Several reports have proposed treatment algorithms that prioritize anticoagulation and non-operative management, reserving surgical thrombectomy for patients who demonstrate inadequate response to conservative management.^5^ The 2020 European Society for Vascular Surgery guidelines recommend considering catheter-directed thrombolysis or open surgical revascularization in infants and children with ALI who fail to improve following initial heparin^6^ therapy (Class 2b; Level C evidence).

An inverse relationship exists between age in the paediatric population and outcomes following invasive interventions for ALI. Evidence indicates that surgical intervention is associated with prolonged hospital stays in infants compared to adolescents and older children.^1^

In the present case, an endovascular approach utilizing mechanical thrombectomy was deemed appropriate, given the patient’s larger-calibre vessels relative to younger paediatric cohorts. The CFA measured approximately 6 mm in diameter, accommodating the 7 Fr sheath (approximately 2.3 mm in diameter) required for delivery of the aspiration thrombectomy device. The procedure was completed, resulting in complete restoration of distal lower limb perfusion. The patient demonstrated prompt recovery with only a minor puncture site wound, reflecting the minimally invasive nature of this approach compared to open surgical intervention.

Compared to catheter-directed thrombolysis, aspiration mechanical thrombectomy offers several advantages, including more rapid thrombus removal due to continuous aspiration, elimination of prolonged catheter dwell time with thrombolytic infusion, reduced bleeding risk, and more complete thrombus evacuation. Additionally, the Penumbra Lightning technology permits differentiation between blood flow and thrombus by adjusting aspiration levels, thereby optimizing blood conservation.

Available data regarding the safety and efficacy of vacuum-assisted thrombo-aspiration systems are predominantly derived from adult populations, particularly elderly cohorts. The evidence base has evolved from single-centre studies^7^ and multicentre retrospective analyses, such as the PRISM trial,^8^ to larger prospective multicentre investigations. Notably, the INDIAN registry, the largest study to date assessing the Indigo Penumbra system in real-world clinical practice, demonstrates that mechanical thrombectomy with the Indigo system is a safe and effective primary modality for revascularization in acute lower limb ischaemia.^9^ This is further corroborated by interim results from the INDIAN-UP trial, which reinforce the safety and efficacy profile of vacuum-assisted thrombo-aspiration systems.^10^

Another prospective, international multicentre study, the STRIDE trial, similarly demonstrated a high rate of successful limb salvage at 30 days with minimal peri-procedural complications^11^ in patients with acute lower extremity ischaemia. The principal challenge of endovascular mechanical thrombectomy in the paediatric population lies in the smaller vessel calibre, which affords a narrower margin for procedural error. The underlying aetiology is also critical; chronic vascular disease in adults is often better tolerated due to the development of collateral circulation, in contrast to the abrupt ischaemic impact of acute thrombus formation in children. Data on endovascular treatment of ALI in paediatric patients remain limited, largely confined to case reports and small series from individual centres. Consequently, management of this population continues to require individualized, multidisciplinary team deliberation to determine the most appropriate therapeutic approach.^12^

## Conclusion

This case report offers anecdotal evidence that may serve as a catalyst for future studies evaluating the feasibility of an endovascular approach using aspiration mechanical thrombectomy in the paediatric population. It highlights the potential to identify a clearly defined subset of patients who may benefit most from such interventions. Our experience provides valuable clinical insight and adds to the growing armamentarium for managing ALI in young children.

## Data Availability

The data underlying this article are available in the article and in its online supplementary material.

## Lead author biography

Raja Muhammad Burhanudeen Afiq Raja Badrol Hisham is a cardiothoracic surgery resident from Malaysia, with a keen interest in both endovascular and open aortic surgery.
